# The Medical Student

**Published:** 1859-01

**Authors:** 


"*The Medical Student.*—The collegiate divides mankind into two great classes—those within the college walls, whether greenys, sophs, juniors, or seniors, are "Fellows," while all the outside world are "Snobs;" and all grades of the former will unite to resent an insult by the latter to the humblest of their number. So when a young man makes choice of the medical profession, and enters its ranks as a student, no matter how humble his position, he forms a constituent part of that profession, and will always find its members ready to stand by and defend him, so long as he is worthy of their support.

"A newspaper of this city—the *City Item*—in a recent article, does manifest injustice to a very worthy class of young men, viz: our medical students. It says:

"'Their education, it must be allowed, is (in the majority of instances) neither finished nor respectable. They will pardon us for so severe a statement; but we make it because it is true, and because we wish to do them some good. It is true. A visit to the lecture rooms of our colleges will prove it to be true. What description of young men are to be seen in these places? Most of them have a Texan Ranger look. Nobody in the world would pronounce them to be refined, liberally endowed young gentlemen. Hair as long as that of a savage, moustaches as fierce as the whiskers of a tiger, a reckless expression of the eye, a long, shuffling, clumsy gait, sword canes, dirk knives, revolvers, attire very unfashionably made, hard swearing, hard drinking, coarse language, cigars, tobacco quids, and pools of tobacco spittle are too prominent barriers for the formation of so flattering a judgment. The picture is not overdrawn. We might make it a great deal less flattering, and then we would be absolutely true.'

"Now, we join issue with the *Item* on the above statements, and say that they are harsh, and destitute of foundation in fact, as regards the large majority of the students. We are willing to admit that there are in our colleges, young men whose early education is sadly deficient, and some whose qualifications better fit them for almost any other pursuit than that of medicine; but to assert that this is true of a majority, or even of any considerable portion of them, we hesitate not to say is very far from true. The writer of that article evidently knows nothing of student life. The student is a genus by himself, and no more the type of the future physician, to the uninitiated, than is the chrysalis of the future butterfly.

"Our medical students are gathered from every section of our widely-extended domain, and from foreign countries. They often journey thousands of miles to get here, and they bring with them the fashions and customs that prevail in their respective neighborhoods, and it is not to be wondered at, that on their first arrival here they should present a somewhat uncouth appearance to the unpractised eye. They are not to be judged of by that abortion of humanity—a Chestnut street dandy. We venture to say that five-sixths of them belong to the most refined and educated classes in their respective neighborhoods, and that there, as here, separated from the peculiar characteristics of "student life," they make a very different appearance from that which they assume while attending lectures. Let our critic witness these same young men when they receive their diplomas next spring, and the following spring, and he would scarcely recognize them—not one bit more than in the editor of to-day he would recognize the "printer's devil" of twenty-five years ago. We venture to say that the editor of the *Item* himself cut but a sorry figure for an editor a score of years back. We know not that he was ever a "devil" himself—he may have been born an editor—but he certainly has room for improving his knowledge of medical students and their habits.

"The assertion that 'medical students are a contemned, despised class,' in this city, is simply untrue, as we can testify from personal observation, and hundreds of others can do the same thing. On the contrary, they are highly respected as a class, and receive very flattering attention from our citizens of all classes. The crowded audiences of the youth and beauty of Philadelphia, that assemble every Spring when the diplomas are conferred, are evidence of the interest felt in them here, and this is but one evidence out of many that might be adduced.

"While depreciating the character of our students, to place them if possible in a still worse light, our critic exalts the attainments of students in foreign countries above the position that is warranted by facts, if we can judge from our foreign medical periodicals. With all their advantages over us, the European schools send out some very unworthy young men. Some very sorry specimens have brought their diplomas to this side of the Atlantic.

"Our own judgment in respect to the students in our colleges this winter—a judgment founded on extended means of observation, on personal contact with them, and on a knowledge of the peculiarities of "studen life," is, that they are, as a whole, a very superior class of young men, and that they will do credit to themselves, to their instructors, and to their chosen profession."

We copy the above from the "*Philadelphia Medical and Surgical Reporter*" for the last week in November. We are glad to have an opportunity to vindicate those who have resolved to embrace our laborious, and frequently thankless profession, and can not but express our indignation at the light in which medical students are regarded in some of our large cities· and especially in that immense vortex of medical instruction, Philadelphia' Instead of being proud of sustaining the two largest medical schools in the world; of sending out yearly hundreds of men, who form the greater part of the most useful and enlightened of professions, and one which can boast of greater practical acumen in this country than in any other; men who go into the wilds of the far west to yearly combat perhaps that terrible scourge, the yellow fever; a profession from whose ranks rose spontaneously the "*Noble Army of Martyrs*" that fell at Norfolk; the general expressions of the citizens of Philadelphia are those of regret that they should be so overrun with ruffians. The hotels have placarded in a conspicuous place, before the entrance to the conveniences of the house "For the use of guests of the house only. *This notice is especially intended for Medical Students.*" That the proprietors of hotels should not desire that their conveniences should be used by every loafer in the street, is very natural and very proper; but we conceive that they have no right to offer such a wanton insult to medical students, who are destined to form perhaps the most respected, and and certainly the most useful class of society. An eminent southern professor who paid a visit to Philadelphia last summer noticed this fact, and commented upon it in no measured terms.

In addition to the pride which a city should feel at presenting such advantages for medical instruction, and in having those advantages so generally appreciated, consider, for a moment, the tangible benefits which result from the yearly presence of such a number of students. First, of course, we will look at it in a pecuniary light. There are probably fifteen hundred medical students in the city of Philadelphia every winter; many of these remaining in the city throughout the year. Calculating that each student spends $110 for instruction, and $250 for his other expenses, we have $540,000 spent in the city of Philadelphia by this despised class. In the face of this simple fact, that a daily paper should publish an article abusive of these visitors, would be in wretchedly bad taste to say the least, granting that all they said was true; and we supposed that the delicacy of a person of manly impulses would have prevented the appearance of such an article as is copied, in part, by the "Reporter." We venture to say that no medical man has felt sensitive at the good natured satire on the medical student in the celebrated character of Bob Sawyer; and there are few who have not exercised their sides over Punch's "Physiology of the London Medical Student." All this is very well and very funny; but we cannot regard some portions of the article quoted above, as any thing but an abusive perversion of the truth; when it says, for example, "Nobody in the world would pronounce them (the medical class) to be refined, liberally endowed young gentlemen," and the writer of that sentence may have repeatedly owed his life, and the lives of those who are nearest to him, to the skill and patient assiduity of men who once belonged to this class; if he has not, he may see the time when he will welcome one of these very men whom he describes with "hair as long as that of a savage, moustaches as fierce as the whiskers of a tiger" etc. etc. like an angel of light. Let the editor of the "*City Item*" think of this article the next time that the life of a near friend depends on the skill and kindness of the good physician.

Let us look at the advantages, in another point of view, which accrue to the city of Philadelphia, from its great number of medical students. The best medical talent in America is drawn there, by the large revenues from the schools. Thus, Philadelphia not only furnishes men, of which we, as a nation, are proud; but these men go to form a society as refined, as cultivated, and as intellectual, as any in the world. It has sent down to posterity names, not only venerated by us, but known by every one. It gives to us most of our valuable text books, and has produced several authors, whose works are used in European schools. Why is all this? It is because nearly $200,000 are annually paid for medical instruction, by *medical students.* In addition to this, men, from all parts of the world being acquainted personally with the most distinguished medical authors, and sitting for months under their teachings, an immense sale is made of medical works, published in

Philadelphia, and support, and employment, is given to hundreds of mechanics, as well as large fortunes to the publishers. All this, too, is due to medical students. These hotels, which put up the placards which we have noticed, come in for their share of the spoils; students put up at them while in search of a boarding-house, and some of them remain there during the entire session; we conceive that no one escapes by paying less than ten dollars at a Philadelphia hotel, and at this low estimate, the proprietors receive from students $15,000.

The medical student is a creature of a peculiar species, whose natural history is not understood by the world at large. His studies are peculiar. His mode of thinking is peculiar. Everything in his pursuits combines to disconnect him from other men. When he commences the study of medicine, has fainted at witnessing his first operation; can eat an apple in the dissecting room, cutting it with his cartilage knife; he is a changed man. He admires to discourse learnedly on the bones to unprofessional hearers; though his style of conversation is materially changed when he is at the "quiz;" he pronounces long names with fearful rapidity and inaccuracy; in short, he wishes to make still more mysterious to the uninitiated, the divine science of medicine. With these mental changes, his person is frequently neglected, especially when engaged in dissecting; he does not go into society, as his pursuits would be discovered by the nose, as well as by the eye and ear. This, as well as an absence cf "metallic tinkling" in the superior femoral region, begets a carelessness of dress, which, perhaps, did not before exist, and will wear off as he emerges the practitioner. As is stated by the editor of the Reporter, students are gathered into Philadelphia from all quarters of the globe; many of them are from the south, bringing their southern garments, and while they may have been most elegantly dressed beaux at home, they do not appear to much advantage on Chestnut street.

Medical students are good fellows, mind their own business, most of them work hard at the profession, smell of the dissecting-room, and chew tobacco. They are of us and with us, and we hold it our duty to defend them.

